# Severe emphysematous pyelonephritis: conservative management plus image-guided percutaneous drainage prior to successful elective nephrectomy. A case report and review of the literature

**DOI:** 10.1259/bjrcr.20140004

**Published:** 2015-05-05

**Authors:** W Tilden, S Valliani, H S Chana

**Affiliations:** Northwick Park Hospital, London, UK; 0000-0002-7166-9639

## Abstract

Emphysematous pyelonephritis (EPN) is a severe necrotizing infection of the kidney, characterized by gas within the renal parenchyma. It is strongly associated with diabetes mellitus and ureteric obstruction. We describe the case of a young female with severe EPN and gas extending diffusely throughout the abdomen. We demonstrate the progression of her infection on CT images from initial admission to percutaneous drainage (PCD) of a large air-fluid collection and then to a period of non-resolution that required elective nephrectomy. Although emergency nephrectomy was historically indicated in EPN, image-guided PCD is emerging as an effective nephron-sparing intervention when combined with supportive care and antibiotic therapy. This can be followed by elective nephrectomy if indicated.

## Clinical presentation

A 27-year-old female of Asian origin was admitted with a short history of generalized abdominal pain, malaise, fever and vomiting. She was clinically shocked, dehydrated and septic with a C-reactive protein of 590 mg l^−1^, creatinine 179 µmol l^−1^, sodium 120 mmol l^−1^ and potassium 3.1 mmol l^−1^. In addition, her blood glucose was over 20 mmol l^−1^ and she had a compensated metabolic acidosis with ketonuria. There was no prior history of diabetes. She was immediately treated for diabetic ketoacidosis secondary to sepsis of unknown origin in a high dependency unit.

## Differential diagnosis

The diagnosis on admission was thought to be non-emphysematous pyelonephritis (EPN) with first presentation of Type 1 diabetes mellitus with diabetic ketoacidosis. A less likely consideration was severe bacterial gastroenteritis.

## Imaging findings

**Figure 1.**
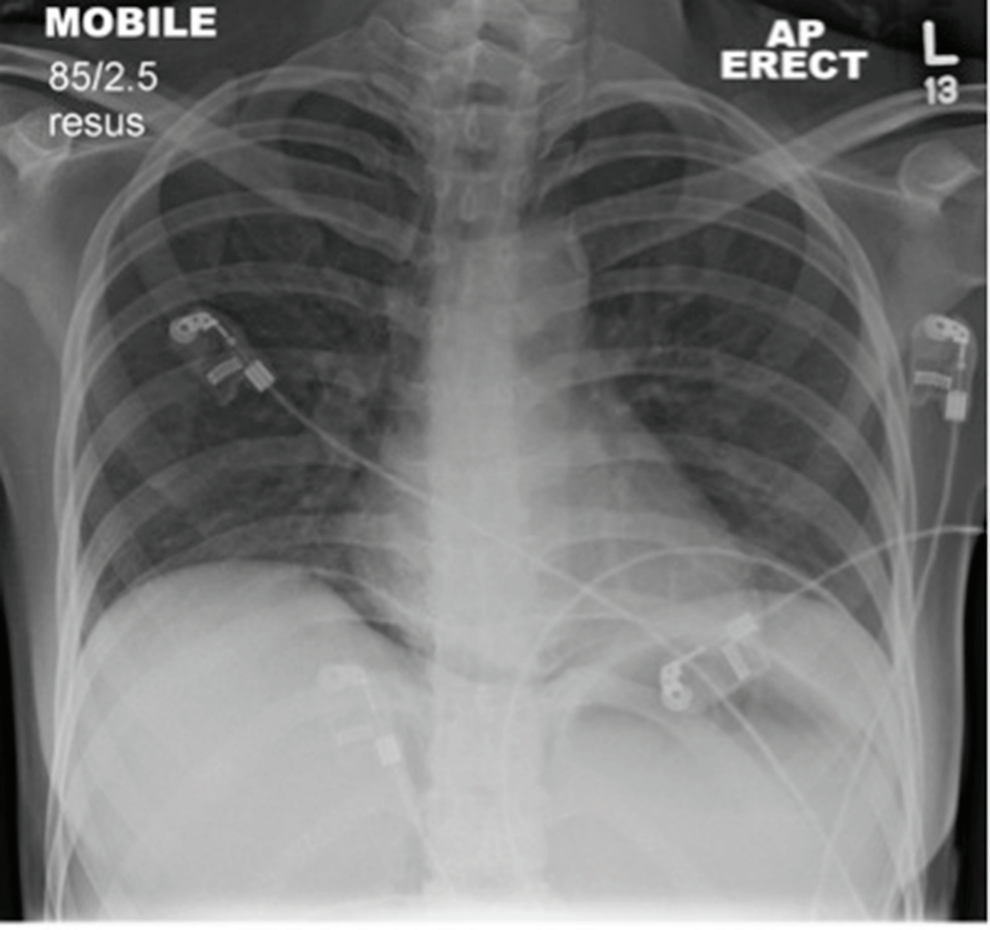
Abdominal film showing well-defined mottled gas density and gas tracking into retroperitoneum.

**Figure 2.**
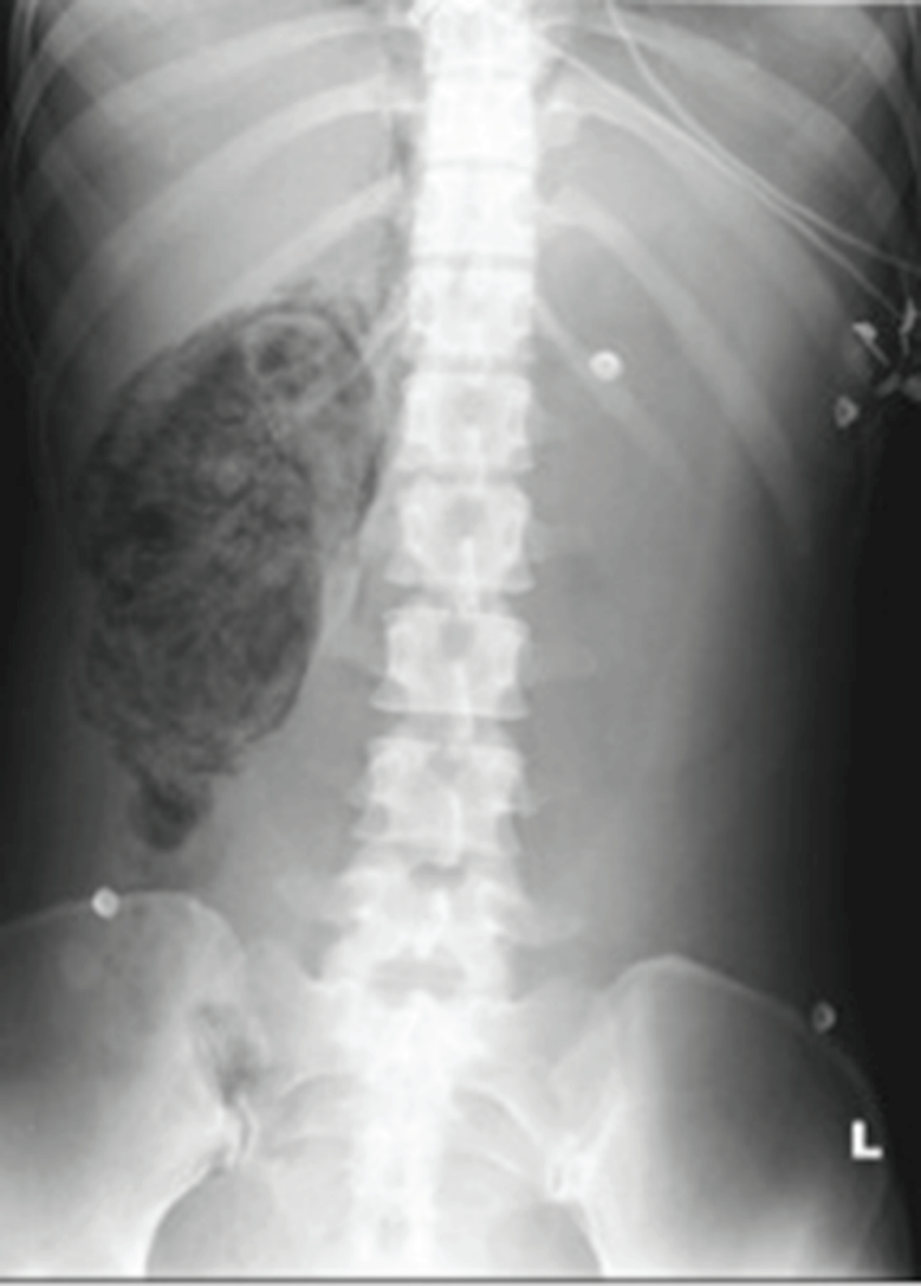
Chest X-ray demonstrating subdiaphragmatic free gas.

**Figure 3.**
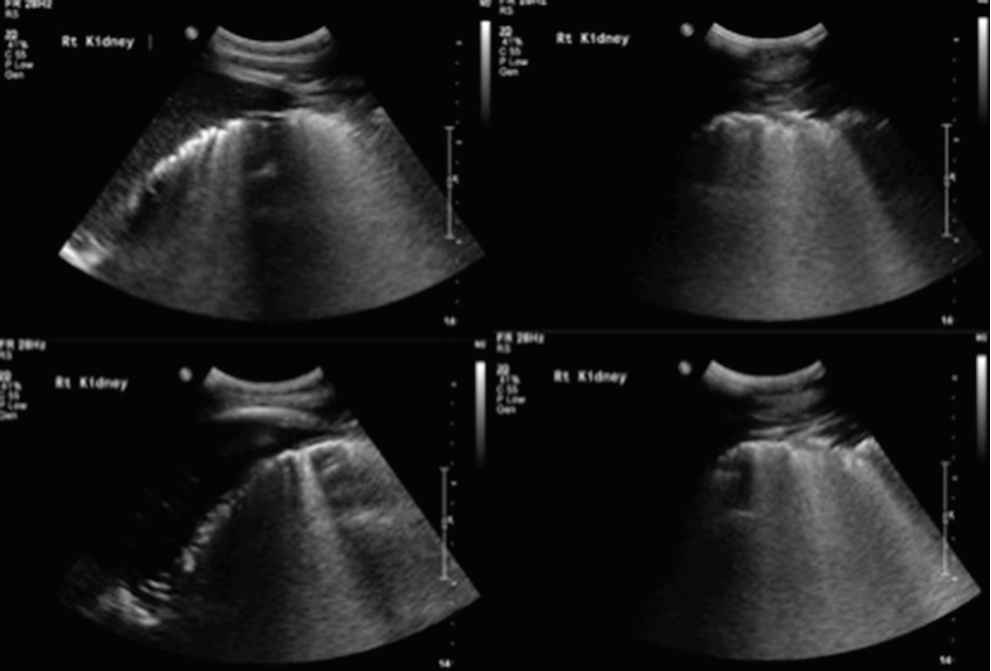
Ultrasound images of right kidney demonstrating the difficulty in obtaining clear images.

**Figure 4.**
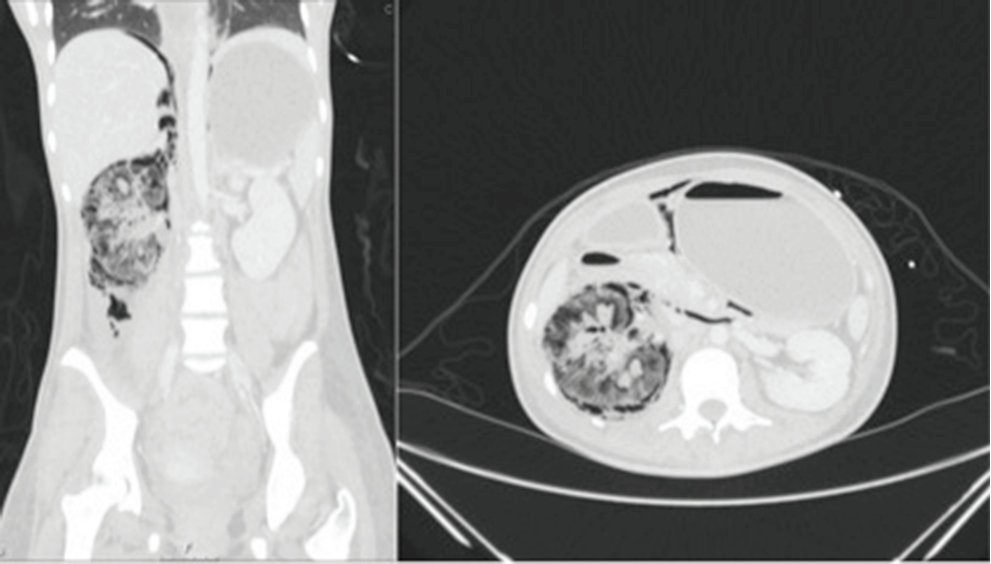
CT image of lung windows showing grossly emphysematous right kidney. Gas is also seen tracking into the retroperiotenum and subphrenic space.

Her abdominal pain worsened and an initial abdominal X-ray was requested. This demonstrated a well-defined right-sided mottled gas density with streaks of gas in the right upper quadrant towards the midline, indicating likely further gas within the retroperitoneum (Figure 1). Additionally, an erect chest X-ray showed subdiaphragmatic free gas (Figure 2). An abdominal ultrasound was of no diagnostic value owing to the significant presence of gas in and around the right kidney (Figure 3). An abdominal CT scan was therefore performed and revealed a grossly abnormal right kidney with gas seen throughout the entirety of its parenchyma, within the right proximal ureter and tracking both superiorly and inferiorly within the retroperitoneal space and possibly within the peritoneum (Figure 4). The diagnosis was severe EPN.

## Treatment

Medical management (MM) with intravenous ertapenem was commenced. The patient was stabilized, and her inflammatory markers and renal function returned to baseline over a period of 2 weeks. She was discharged and returned to the ambulatory unit for daily administration of intravenous antibiotics.

**Figure 5.**
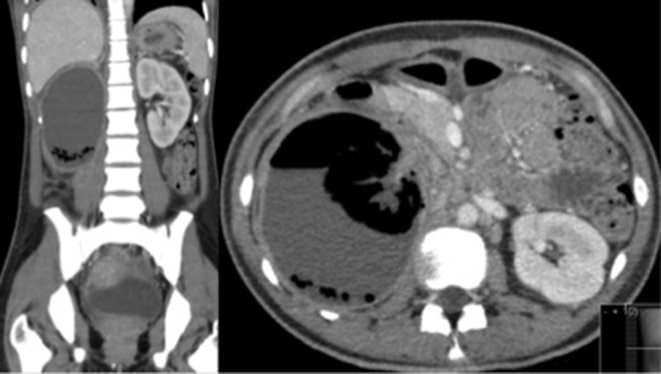
CT image of abdominal windows demonstrating persistent emphysematous change with new 11 × 9 × 12  cm air-fluid collection around the right kidney.

2 weeks later, the abdominal pain recurred and the patient was re-admitted. A repeat CT scan demonstrated persisting emphysematous change in the right kidney, with a new large gas- and fluid-containing collection in the right pararenal space (Figure 5). This was considered amenable to percutaneous drainage (PCD), which was performed under ultrasound guidance with good clinical resolution. The specimen grew extended spectrum beta-lactamase-producing *Escherichia coli* sensitive to ertapenem.

## Outcome and follow-up

Although the fluid collection had reduced in size, draining over 10 ml of fluid per day over an extended follow-up period while on intravenous antibiotics, the complex fluid collection persisted on follow-up CT scan, supporting the decision to proceed with elective nephrectomy.

She underwent an elective open radical right nephrectomy a total of 6 months after her first presentation with EPN. There were no complications and she remained well 2 weeks post surgery with normal inflammatory markers and renal function.

## Learning points

EPN is a rare condition, with a 2008 systematic review of 10 retrospective studies reporting a total of only 210 cases in the literature.^1^ Most cases present in the fourth or fifth decade and there is a female preponderance of 6 : 1, with a high proportion of these individuals being of Asian origin.^2^

The main presenting features are similar to regular pyelonephritis—dysuria, nausea, vomiting, flank pain and fever—the diagnosis can therefore often be missed clinically.^2^ Early radiological investigation is essential owing to the potential for rapid progression to septic shock. Several imaging modalities, including ultrasound and plain radiograph, can make the diagnosis, but CT scan is preferred with its increased sensitivity and use in assessing the extent of parenchymal damage in EPN, as well as detecting stones in obstructive cases.^2^

There are several classification systems for EPN; however, the most widely used systems are those of Wan et al^3^ and Huang and Tseng^4^, who determined prognostic indicators based on CT scan appearances (Tables 1 and 2). Wan et al Type I is associated with a poorer prognosis, as are Huang and Tseng Type 3 and 4.

**Table 1.** Wan et al.^3^ classification systems for EPN.

Type I	Type II
Parenchymal necrosis	Parenchymal necrosis
Gas present	Gas present
No fluid	Fluid in the renal parenchyma, perinephric space or collecting system

EPN: Emphysematous pyelonephritis.

**Table 2.** Huang and Tseng^4^ classification systems for EPN.

Type 1	Gas within the collecting system
Type 2	Gas within the parenchyma
Type 3	Gas outside the kidney a: in the perinephric space b: in the pararenal space
Type 4	Bilateral EPN or gas in a solitary kidney

EPN: Emphysematous pyelonephritis.

Historically, EPN has been managed with emergency nephrectomy or open surgical drainage with antibiotic therapy, the anticipation being that delayed nephrectomy led to increased mortality rates. This carried a mortality rate of 40–50%.^5^ As image-guided interventions have become more advanced and accessible, this approach has now evolved and the focus is on salvage of the renal unit. Indeed, Aswathaman et al^6^ found that those who did not undergo nephrectomy had a mean relative renal function of 42% in the affected kidney after resolution of the acute episode on functional nuclear imaging.

In addition, Kapoor et al^7^ retrospectively analyzed outcomes of 39 patients presenting to their unit with EPN, finding that emergency nephrectomy was associated with a substantially higher mortality rate than MM plus PCD—43% *vs* 8%. They also found that all patients who underwent delayed nephrectomy after failed MM plus PCD had survived. This tallies with a systematic review by Somani et al^1^ of 210 patients presenting with EPN. They found that conservative management alone was associated with a mortality rate of 50%, whereas the mortality after emergency nephrectomy plus MM was 25%. The mortality for PCD plus MM, however, was just 13.5%. They also found that those who underwent PCD plus MM and then went on to have elective nephrectomy had an even lower mortality rate of 6.6%. The same authors noted that systemic shock and a creatinine level of over 140 µmol l^−1^ was associated with higher patient mortality. Our case describes a particularly fulminant course of EPN. The patient initially had poor prognostic features in that she was shocked and had an elevated creatinine. Her initial CT scan appearances also demonstrated Wan et al Type I EPN and Huang and Tseng Type 3B EPN, corresponding with the poorest possible prognosis. Despite this, clinical success was attained through initial MM and supportive care followed by ultrasound-guided PCD and eventually elective nephrectomy. This case therefore supports the evidence that PCD and MM should be considered over early nephrectomy in EPN, even in patients with very poor clinical and radiological prognostic factors.
